# A new *Mylabris* species from south-eastern Iran and a key to the Iranian species of the nominate subgenus (Coleoptera, Meloidae)

**DOI:** 10.3897/zookeys.219.3674

**Published:** 2012-09-04

**Authors:** Sayeh Serri, Zhao Pan, Marco A. Bologna

**Affiliations:** 1 Insect Taxonomy Research Department, Iranian Research Institute of Plant Protection, Tehran, 19395-1454, Iran; 2 Dipartimento di Biologia Ambientale, Università Roma Tre, Viale G. Marconi 446, 00146 Roma Italy

**Keywords:** Iran, taxonomy, new species, key to the species

## Abstract

A new species of *Mylabris* of the nominate subgenus is described and figured. This species is apparently endemic to the south-eastern Iranian province of Kerman and seems to be phenetically very distinct from all other species of this subgenus, primarily because of the unique elytral pattern. A key to the species of the nominate subgenus distributed in Iran is also presented.

## Introduction

The Meloidae of the Middle East remain poorly studied even though this area is one of the most interesting of the Palaearctic region from biogeographic and taxonomic points of view. Within the Middle East, the family is most speciose in Iran which harbours a fauna with several endemics together with many species in common with Central Asia, Anatolia and Levant, as well as some Saharo-Sindian and Arabic components in the southern provinces. The descriptions of species and faunistic records concerning the blister beetles of this area are few and highly scattered in the literature. The only synthetic works on the Iranian Meloidae were published in the last century by Kaszab (1957; 1968) and Mirzayans (1970). Bologna (2008), in the Palaearctic catalogue of Meloidae, listed 194 species for this country.

The Hayk Mirzayans Insect Museum (HMIM) of the Iranian Research Institute of Plant Protection and the M. Bologna collection at the University Roma Tre (CB) house several new species of Iranian Meloidae of the subfamilies Nemognathinae and Meloinae. Unresolved taxonomic problems prevent all from being described at this time. However, we are taking this opportunity to describe one very distinct new *Mylabris* Fabricius, 1775 belonging to the nominate subgenus. It is endemic to a narrow area of Iran and represents the southeasternmost species of the nominate subgenus except for *Mylabris quadripunctata* (Linnaeus, 1767) and *Mylabris variabilis* (Pallas, 1781), which are broadly distributed from the Iberian peninsula to western China.

The meloine genus *Mylabris* is easily distinguished using a key published by Bologna and Pinto (2002), and the nominate subgenus was well defined by Kuzin (1954) and Pardo Alcaide (1950). The most recent comprehensive key to the species of *Mylabris* remains that published by Sumakov (1915). In order to help with the identification of the Iranian species of the subgenus *Mylabris*, we provide a key to its species recorded in Iran.

## Results

### Mylabris (Mylabris) barezensis sp. n.

urn:lsid:zoobank.org:act:0141DDB7-3BC1-49EC-A502-776A5004B625

http://species-id.net/wiki/Mylabris_barezensis

Figure 1

#### Type specimens.

Holotype male (HMIM), and 2 males (CB) and 1 male and 2 female paratypes (HMIM), labelled “ Dehbakri, 6.5.1969, Paz.& Hasch.”.

#### Type locality.

**IRAN**, Dehbakri (29.0539°N, 57.9131°E); an Iranian town in the Kerman province, about 55 km southwest of Bam, east of Jebal Barez mountain, at 2027 m a.s.l. This mountainous area was characterized by forest until before the second World War (Planhol 1969) but now secondary steppe habitats are widely spread.

#### Diagnosis.

**Figures 1–8.**
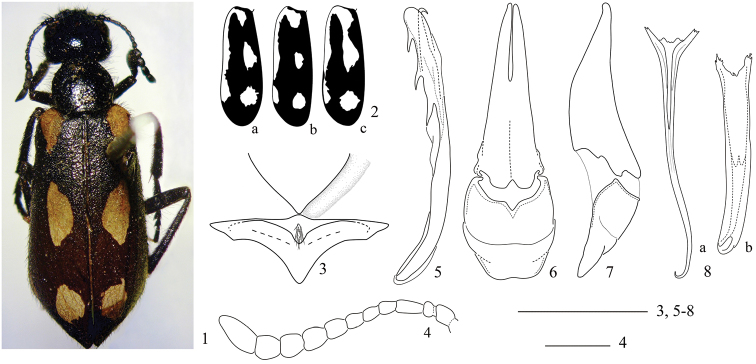
*Mylabris barezensis* sp. n. **1** Habitus **2a–c** variation of elytral pattern **3** mesosternum and mesepisterna **4** male left antenna **5** aedeagus, lateral view **6** tegmen, ventral view **7** tegmen, lateral view **8a–b** variation of *spiculum gastrale*. Bar scales: 1 mm**.**

A *Mylabris* species belonging to the nominate subgenus as defined by Pardo Alcaide (1950), Kuzin (1954) and Bologna (1991). Immediately distinguishable from other species of the subgenus by the unique elytral pattern characterized by reduced brown-orange surface and the large extension of black colouration everywhere as Figs 1 and 2. Male gonoforceps in lateral view (Fig. 7) slender. The mesosternum (Fig. 3) is narrowed posteriorly and the setae on its anteriorly modified section are longer than usual.

#### Description.

Body uniformly black, except elytra which have the following pattern: black colouration largely extended everywhere, except brown-orange as follows: at base (along the scutellum excluded), along the external margin except at apex (Figs 1, 2a) (in one specimen the posterior section of the external margin is fragmented in one spot as in Fig. 2b) and with two sub-oval spots, one in the middle, and another on the posterior third; in one specimen, the middle spot is fused to the brown-orange base (Fig. 2c). Setation uniformly black, but ventral side of male foretibiae and foretarsi with golden setae, forming a small pad under the pro- and mesotarsomeres; setation evidently longer on venter than dorsally; setae denser on head and pronotum, sparser on elytra. Body length: 10–15 mm.

Head slightly longer than wide at temples level (excluding mandibles), wider at temples than at eyes; punctures relatively deep, large and irregular, surface among punctures shagreened, shiny on vertex, wrinkled on frons; head capsule subquadrate, temples broadly curved posteriorly and subequal in length to the longitudinal length of eye; frons flat, in the middle with one red spot more or less divided posteriorly; clypeus transverse, convex, with slightly rounded anterior and lateral margins, anteriorly depressed, fronto-clypeal suture clearly visible; labrum only slightly shorter and narrower than clypeus, anterior margin sinuate, longitudinally depressed in the middle; mandibles robust, curved, in lateral view longer than clypeus and labrum together; maxillary palpomere II with very long setae on the posterior side, last maxillary palpomere apically thickened and truncate at apex; antennae (Fig. 4) extending almost to posterior margin of pronotum in male, scapus more than twice as long as pedicellus, pedicellus semi-globular; antennomere III elongate and about 1.2 times as long as IV–V together, IV and V similar in length, VI similar in length to IV and V but slightly widened apically, VIII – X progressively more elongate and apically widened, X subcylindrical, last antennomere elongate and narrowed in the last third, particularly in male.

Pronotum slightly wider than long, narrowed anteriad, convex, without evident depressions or with a superficial rounded depression in the middle of each side, maximum width posterior to middle; punctures almost confluent on anterior and posterior third; elytral pattern as in Fig. 1 and Fig. 2 (a, b, c), elytral setation shorter and sparser than that on head and pronotum, erect on the anterior third, recumbent and shorter on the remaining surface; mesosternum longitudinally elevated in the middle, with a clearly modified anterior section (“scutum”), with a slightly depressed oval area with dense and very long setae (Fig. 3); mesepisterna depressed along the anterior margin, which consequently appears to be raised. Legs black, pro- and mesotibial spurs both similar in shape and pointed, the inner metatibial spur stick-like and the external one pointed; femora with mixed short and long setae, setae robust and more elongate on tibiae and tarsi; male foretibiae ventrally with mixed golden and black short and dense setae, in female with short dense black pubescence and also elongate black setae on external side; male pro- and mesotarsomeres with ventral golden setae forming tarsal pads, those of mesotarsomeres smaller.

Sternite VIII deeply emarginate at middle of posterior margin in male, rounded in female. Male genitalia as in Figs 5–8: in lateral view (Fig. 7) the basal part of gonoforceps slender, apical lobe of gonoforceps relatively short, slightly less than half the length of total gonoforceps; in ventral view, gonoforceps as in Fig. 6; aedeagus (Fig. 5) with two distinct subequal hooks, both positioned far from apex and with the same inclination, the proximal one slightly longer; endophallic hook slender and curved (Fig. 5). The apodeme of the s*piculum gastrale* is variable: most males have this sclerite slender in the middle and clearly narrowed and elongate in the last portion (Fig. 8a) but in a single male it is very wide medially (Fig. 8b).

#### Etymology.

This new species is named after the Jebal Barez mountain range in Kerman Province, in the north west of which the new species was collected.

#### Taxonomic remarks.

The taxonomic revision of the nominate subgenus of *Mylabris* as well as its phylogenetic study remain in preparation (Pan et al. unpublished). The nominate subgenus at present contains 19 species (Bologna 2008), but at least another four undescribed species are recognized and will be described in a systematic study that includes both morphological and molecular data (Pan etal. unpublished).

For this reason we are unable to discuss in detail the phylogenetic relationships of *Mylabris barezensis*. This species is clearly distinct from all others of the nominate subgenus by the unique elytral pattern, as well as by the mesosternal “scutum” with dense and very long setae.

Phenetically, *Mylabris barezensis* is similar to two species of the subgenus *Micrabris* from Afghanistan, namely *afghanica* Kaszab, 1953 and *marakensis badakhaskanica* Kaszab, 1958 (both figured in Kaszab 1958). Similarity is due to the extended black elytral pattern. From both species it can be easily distinguished by setose area on the mesosternum, the wider pronotum, and the shape and position of aedeagal hooks (see Kaszab 1958 for comparison).

The type specimens were previously identified by Zoltán Kaszab, the late Hungarian specialist of Meloidae, as “*Mylabris biguttata* Gebler”, a species which actually belongs to the mylabrine genus *Hycleus*, but has a similar elytral pattern.

##### Key to the Iranian species of *Mylabris (Mylabris)*

**Table:** 

1	Elytral apex with a very wide black fascia, more or less sinuate anteriorly and extended to cover ca. 1/6 of the entire surface	2
–	Elytral apex uniformly reddish-brown or with a narrow black colouration which sometimes extends on the sutural inner margin	6
2	Elytral pattern characterized by reduced orange-brown areas and a large extension of black colouration everywhere	*Mylabris (Mylabris) barezensis* sp. n.
–	Elytral pattern reduced to distinct spots or fasciae, never fused or coalescing to form an extended black network, which leaves isolated orange-brown areas	3
3	Pronotum with a shallow anterior transverse depression. Frons without red macula (exceptions are very rare). Elytra usually with two series of spots, one anterior and one on the middle, and an apical fascia widely emarginate just before the suture (in some populations middle spots fused to form a wide fascia). Aedeagal distal hook long and curved, the proximal one very long and curved forward, longer than distal, positioned far from apex; gonostyli broadly cylindrical, suddenly narrowed apically in two long and slightly curved lobes	*Mylabris (Mylabris) quadripunctata* (Linnaeus, 1767)
–	Pronotum without an anterior transverse depression. Frons with red macula. Elytral pattern not as above. Aedeagal distal hook short, the proximal one clearly longer than distal, both positioned close to apex and more rectilinear; gonostyli uniformly slender, cylindrical	4
4	Elytral pattern with two anterior oblique spots, one middle transverse incomplete and sinuate fascia, neither reaching the external margin nor the suture, and an apical small fascia sinuate on fore margin, not widely extended anteriorly. Aedeagal distal hook only slightly shorter than proximal, both hooks slightly inclined	*Mylabris (Mylabris) parumpicta* (Heyden, 1883)
–	Elytral pattern usually with three fasciae (anterior, middle and apical), the middle one reaching both external margin and suture, the apical one widely extended anteriorly, with fore margin slightly emarginated lateral to the suture (in some populations the anterior fascia is reduced to two spots). Aedeagal distal hook clearly shorter than proximal hook, both clearly inclined	*Mylabris (Mylabris) variabilis* (Pallas, 1781)
6	Antennomeres III-XI (particularly VII-XI) reddish, XI almost brownish. Black apical fascia of elytra very narrow,extended along the suture and reaching the internal margin of subapical transverse fascia	*Mylabris (Mylabris) olivieri* Billberg, 1813
–	Antennomeres uniformly black. Elytral pattern not as above	7
7	Elytra uniformly reddish-brown with an extremely narrow black fascia on the apex	*Mylabris (Mylabris) apicenigra* Soumakov, 1915
–	Elytra uniformly reddish-ochre	*Mylabris (Mylabris) concolor* Marseul, 1870

## Supplementary Material

XML Treatment for Mylabris (Mylabris) barezensis

## References

[B1] Bologna MA (1991)Coleoptera Meloidae, Fauna d’Italia. XXVIII.Calderini, Bologna, 541 pp.

[B2] Bologna MA (2008) New nomenclatorial and taxonomic acts, and comments, Meloidae; family Meloidae Gyllenhal, 1810. In: Löbl I Smetana A (Eds). Catalogue of Palaearctic Coleoptera 5.Apollo Books, Stenstrup, 45–49: 370-412

[B3] Bologna MA Pinto JD (2002) The Old World genera of Meloidae (Coleoptera): a key and synopsis. Journal of Natural History 36: 2013-2102 doi: 10.1080/00222930110062318

[B4] Kaszab Z (1957) Neue Meloiden aus Iran (1954) (Coleopt.). Jahreshefte des Vereins für Vaterländische Naturkunde in Württemberg 112: 50-59

[B5] Kaszab Z (1958) Die Meloiden Afghanistans (Coleoptera). Acta Zoologica Academiae Scientiarum Hungaricae 3: 245-312

[B6] Kaszab Z (1968) Contribution à la faune de l’Iran, 8. Coléoptères Meloidae. Annales de la Société Entomologique de France (NS) 4: 749-776

[B7] Kuzin VS (1954) K posnanyju systemy narybnikov (Coleoptera, Meloidae, Mylabrini). Trudly Usesojnogo Entomologizesko Obzestva 44: 336-379 [In Russian]

[B8] Mirzayans H (1970) Contribution à la connaissance de la faune des Clerides er Méloides de l’Iran. Entomologie et Phytopathologie Appliquées 29: 25-38

[B9] Pardo Alcaide A (1950) Estudios sobre Meloidae. II. Los Mylabrini de la peninsula Iberica, Boletin de Patólogia vegetal y de Entomologia Agricola 17: 61-82

[B10] Planhol X (1969) Le déboisement de l’Iran. Annales de Géographie 78: 625-635 doi: 10.3406/geo.1969.15948

[B11] Soumacov GG (1915) Les espèces paléarctiques du genre *Mylabris* Fabr. (Coleoptera, Meloidae). Horae Societatis Entomologicae Rossicae 42: 1-71

